# A104 ATYPICAL PRESENTATION OF HSD3B7 DEFICIENCY

**DOI:** 10.1093/jcag/gwad061.104

**Published:** 2024-02-14

**Authors:** M Morrissey, P Kawada

**Affiliations:** University of Alberta, Edmonton, AB, Canada; University of Alberta, Edmonton, AB, Canada

## Abstract

**Background:**

Bile acid synthesis disorders are the result of deficient activity of one of 17 enzymes required for conversion of cholesterol into bile acids. One such bile acid synthesis disorder is 3β-hydroxy-Δ5-C27-steroid oxidoreductase (HSD3B7) deficiency, an autosomal recessive inherited disorder caused by a defect in 3β-hydroxy-Δ5-C27-steroid dehydrogenase (C27 3β-HSD) enzyme. Ultimately, this bile acid synthesis disorder is due to a HSD3B7 gene mutation. With only 53 cases reported between 1993 and 2015, HSD3B7 deficiency is rare. Additionally, the existing literature is primarily in the form of case reports, with the majority of patients with HSD3B7 deficiency presenting in the neonatal period, often with cholestatic jaundice.

**Aims:**

To showcase an atypical presentation of HSD3B7 deficiency, in an 11 year old male with recurrent epistaxis and a coagulopathy of vitamin K deficiency. Additionally, to complete a review of the literature available on HSD3B7 deficiency.

**Methods:**

Case report, chart review, and literature review.

**Results:**

11 year old male who initially presented to Pediatric Hematology with prolonged PTT and INR in the context of recurrent epistaxis and easy bruising. Upon further work up, the patient was found to have low levels of vitamin K dependent factors, as well as low vitamin levels of A, D, and E. Additionally, the patient had elevation of AST, ALT, GGT, ALP, and total bilirubin. Normalization of the INR and PTT were achieved with vitamin K supplementation. Genetic testing was sent to work up the abnormal liver enzymes and revealed HSD3B7 deficiency. Fast atom bombardment ionization was subsequently completed and confirmed the diagnosis.

**Conclusions:**

There is limited clinical data available regarding HSD3B7 deficiency. The classic presentation of this bile acid synthesis disorder is in a neonate with cholestatic jaundice, hepatomegaly, fat-soluble vitamin deficiencies, and lipid malabsorption. Therefore, the initial presentation of an 11 year old with recurrent epistaxis and prolonged INR and PTT is thus an atypical presentation of HSD3B7 deficiency. This case report demonstrates that there is diversity in the age of presentation and accompanying signs and symptoms in HSD3B7 deficiency.

**Funding Agencies:**

None

